# Supplementary figures and images for: Do Malassezia yeasts colonize the guts of people living with HIV?
Source: PLoS One. 2025 May 21;20(5):e0322982. doi: 10.1371/journal.pone.0322982 (PMC12094740; doi:10.1371/journal.pone.0322982)

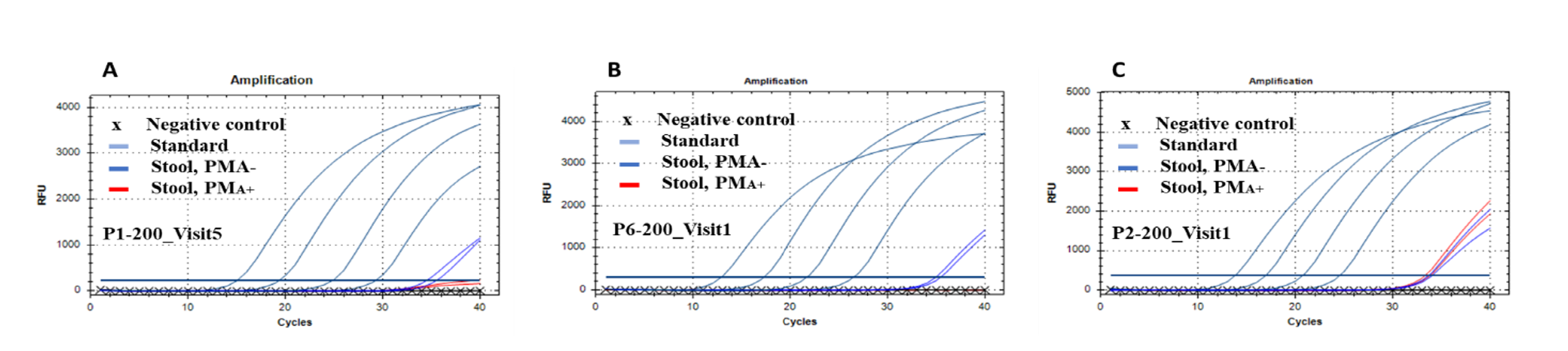

Supplement: Fig S1 — Blue and red curves represent untreated (PMA-) and treated (PMA+) stools, respectively. In panels A and B, the PCR amplified only the untreated samples and not the PMA-treated samples, indicating the presence of dead Malassezia yeasts. In panel C, both PMA-treated and untreated samples were similarly amplified, indicating the presence of living Malassezia yeasts in the sample. (TIF) [file pone.0322982.s001.TIF]

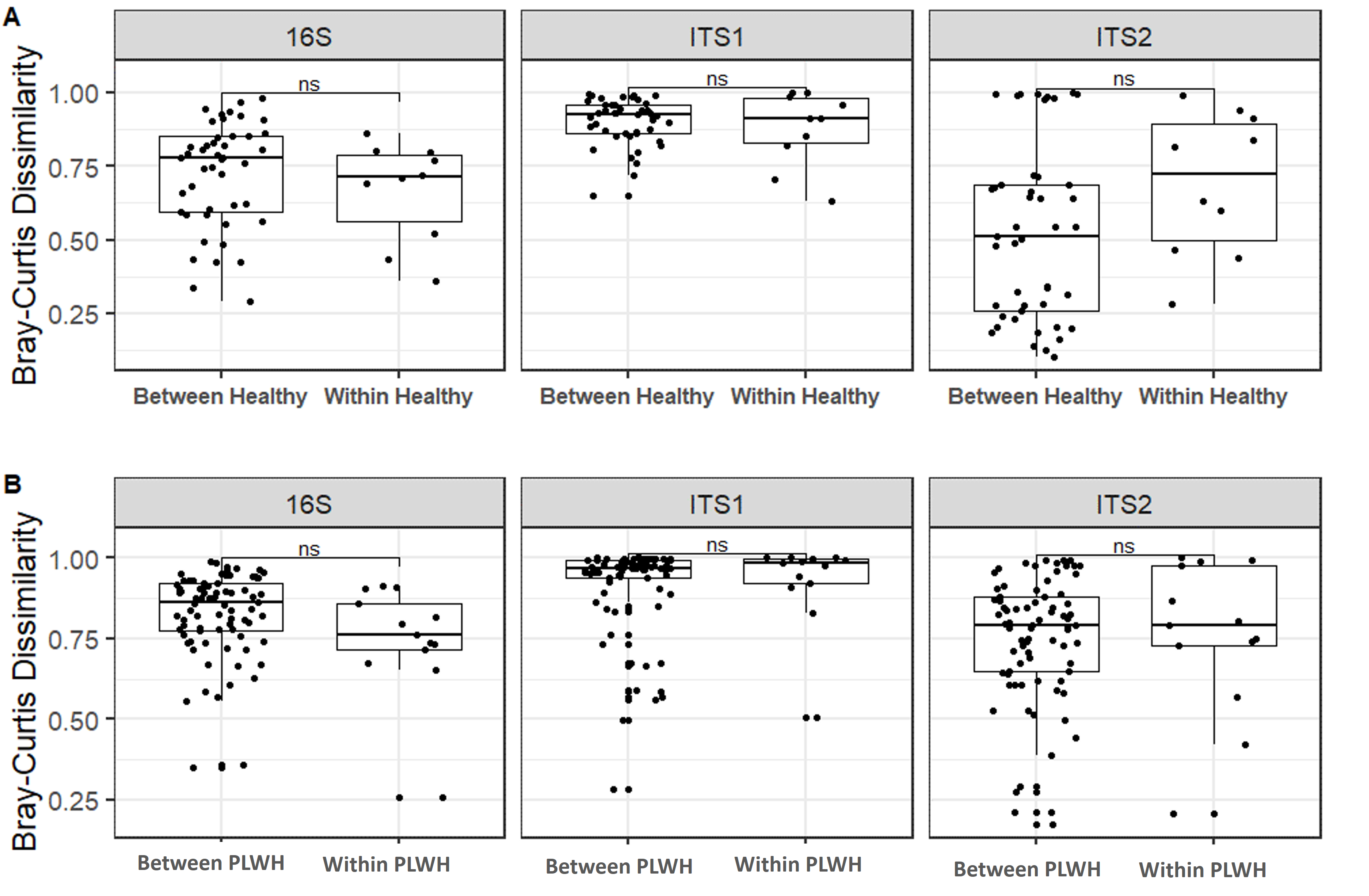

Supplement: Fig S2 — Comparisons of Bray-Curtis dissimilarity values between Visit 1 and Visit 2 samples donated by different participants (A) (between controls, n = 10) or different patients living with HIV (PLWH B) (between PLWH, n = 13) and between samples donated by the same control (within healthy) or the same PLWH (within PLWH) for 16S and ITS. Bray-Curtis dissimilarity values range from 0 to 1, with 0 being the least dissimilar and 1 being the most dissimilar. ns: not statistically significant Wilcoxon test (p > 0.05). (TIF) [file pone.0322982.s002.TIF]

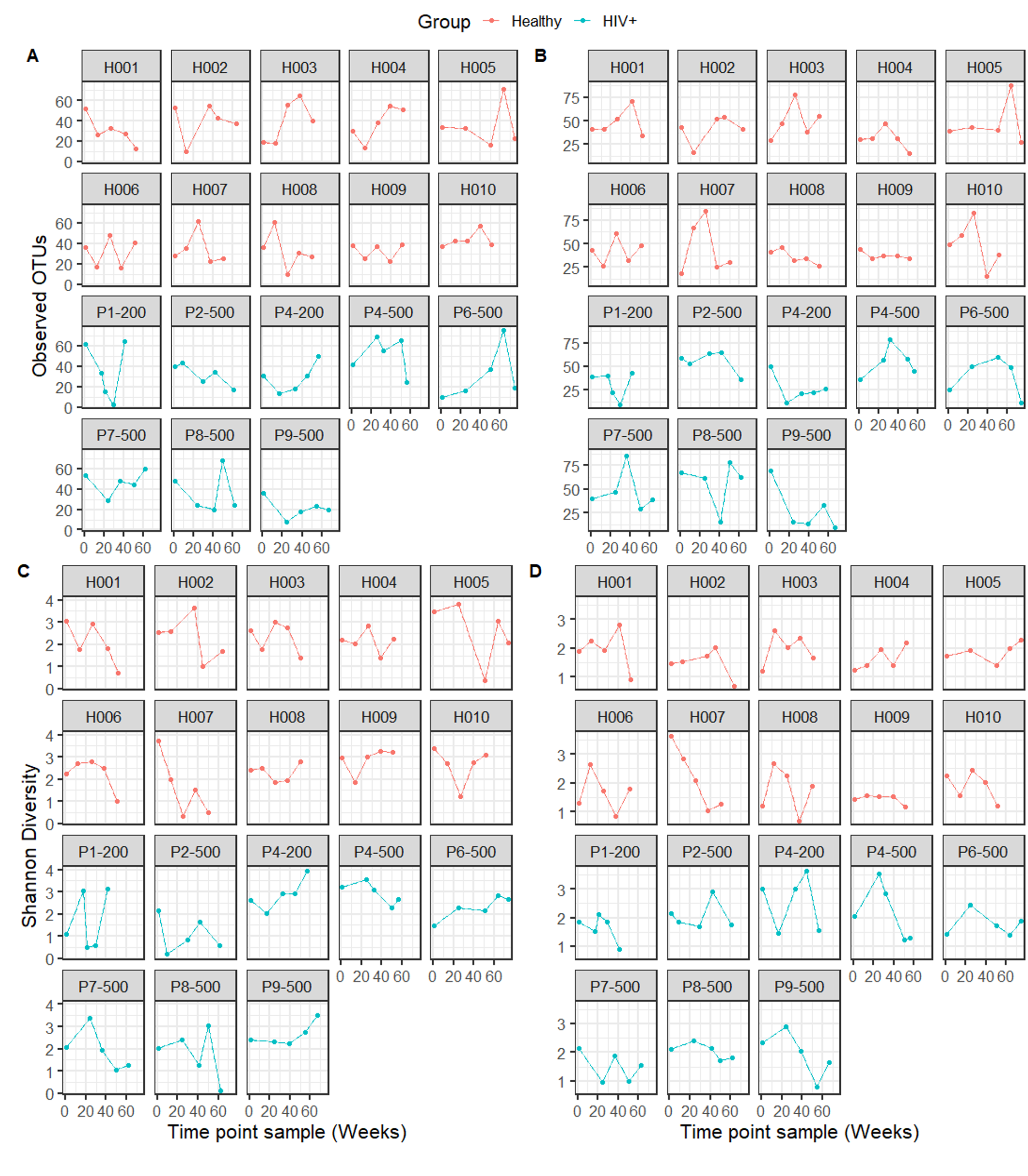

Supplement: Fig S3 — Detail of the observed OTUs (A, B) and Shannon diversity indices (C, D), by ITS1 (A, C) or ITS2 (B, D) metabarcoding, respectively. (TIF) [file pone.0322982.s003.TIF]
